# Noise and Public Hospitals

**Published:** 1912-03-02

**Authors:** 


### The Hospital

The Workers' Newspaper of Administrative Medicine and Institutional Life, Administration, National Insurance and Health.

No. 1338, Vol. LI. SATURDAY, MARCH 2nd, 1912. PRICE ONE PENNY.

### NOISE AND PUBLIC HOSPITALS.

The violent denunciations against street noises which are made from time to time do not seem to have brought about any amelioration of the nuisance which has so often been complained about. Among the chief sufferers from the excess of noise which our modern civilisation has tended to create in street and public thoroughfares are the patients who are in public hospitals, especially those institutions which are environed by commercial activity. In some of these cases the hospitals have existed long before the noises came into being; in a few cases, however, the hospital authorities are responsible for the situation that exists at present, since they have, in spite of all precepts to the contrary, built their institutions on sites which were utterly unsuitable to receive a modern hospital. Among the first category is to be placed one of our London hospitals that was established many years ago. After its establishment a portion of the neighbouring property was acquired by a railway company for the purpose of building a suburban branch line. This branch line was built, and built in such a way that an inconvenient angle was formed close to the hospital grounds, necessitating the use of extra precautions, such as whistling, by the engine drivers. The result of this is that the occupants of the hospital wards, which almost directly overlook the railway, are hourly irritated by the shrieking of the engines. Expostulation has been of no avail in mitigating this nuisance; the railway authorities state that the safety of the public is best secured by prohibiting engine drivers from passing that point without opening their steam whistles to the widest possible extent. In another case—this time a provincial hospital—the institution was built more than fifty years ago. Three years ago came the expansion of the municipal activities in the shape of an extension of the tram lines, which now run past the hospital, causing a considerable disturbance, since trams run for the greater part of the night. The authorities of the hospital have attempted to persuade the civic authorities to meet them at least so far as to pave the entire road in front of the institution with wood paving; the suggestion has been ruled out of court on the ground of expense. In both these cases the hospital suffers from what may legitimately be described as a nuisance, although it may be difficult to prove that the noise really constitutes what lawyers term a nuisance. There is therefore no legal remedy. The only way open to the hospital authorities is to exercise whatever influence they may possess with either railway directors or town councillors to induce these latter to consider some means of remedying the grievance, for a grievance such things undoubtedly are. And there can be little question that in the majority of cases it is an easily removable grievance. Ninety per cent. of our street noises are unnecessary. With that percentage of them at least the authorities should deal, and deal trenchantly, in appropriate by-laws such as are in force in some American cities. Pending this salutary action on the part of municipalities and borough councils, what can the hospitals do?

There is reason to believe that those who vent superfluous energies or express their superabundant vitality and good spirits in the neighbourhood of hospitals by creating a degree of objectionable noise, do so simply because they imagine that the street is the proper place for such tumult. Most of them are unaware that they are within hearing distance of any hospital, and we are sufficiently charitable to believe that if they knew that they were disturbing sick people they would be the first persons to enjoin quietness. The public always respects the request for peace on the part of the sick. Is it too much to hope that what the carman does on occasion for a private patient when straw has been laid down in the street, he may be induced to do as a measure of routine for the in-patients in our hospitals? The experience of the American hospitals proves that this hope is not an idle one. In New York and other large cities one of the first things that strike the attention of the visitor to a hospital is the multiplicity of the warning placards which in large red readable letters bear the inscription, "Please slow down your cart when passing the hospital," "Please make as little noise as possible in the neighbourhood of the hospital," "Please remember that the hospital is in this street and do not blow your horn," or other similarly worded and equally plain requests. It is gratifying to find, on inquiry, that since this policy of appealing to the man in the street has been adopted the percentage of street noises in the vicinity of the hospitals has much decreased.

This, then, is one way in which hospital workers may attack the problem of street noises. Another is by inducing the civic authorities to pave the streets in the immediate neighbourhood of the institution with wood blocks. Where tram lines run such wood paving is essential, and in a residential quarter it is usually laid down at once. The main thing is that the paving must extend for a considerable distance; it is no manner of use paving mere strips in front of the hospital gates. Yet a third method is to make the neighbours of the hospital realise that quietness is a thing greatly to be desired, and to get them to help the institution in securing that desirable essential. With these three methods to begin with, hospitals may do much to lessen the nuisance of noise until that desirable consummation—the introduction of suitable municipal by-laws.

